# Varying solvent type modulates collagen coating and stem cell mechanotransduction on hydrogel substrates

**DOI:** 10.1063/1.5111762

**Published:** 2019-09-30

**Authors:** Alice E. Stanton, Xinming Tong, Fan Yang

**Affiliations:** 1 Department of Bioengineering, Stanford University, Stanford, California 94305, USA; 2 Department of Orthopaedic Surgery, Stanford University, Stanford, California 94305, USA

## Abstract

Type I collagen is the most abundant extracellular matrix protein in the human body and is commonly used as a biochemical ligand for hydrogel substrates to support cell adhesion in mechanotransduction studies. Previous protocols for conjugating collagen I have used different solvents; yet, how varying solvent pH and composition impacts the efficiency and distribution of these collagen I coatings remains unknown. Here, we examine the effect of varying solvent pH and type on the efficiency and distribution of collagen I coatings on polyacrylamide hydrogels. We further evaluate the effects of varying solvent on mechanotransduction of human mesenchymal stem cells (MSCs) by characterizing cell spreading and localization of Yes-Associated Protein (YAP), a key transcriptional regulator of mechanotransduction. Increasing solvent pH to 5.2 and above increased the heterogeneity of coating with collagen bundle formation. Collagen I coating highly depends on the solvent type, with acetic acid leading to the highest conjugation efficiency and most homogeneous coating. Compared to HEPES or phosphate-buffered saline buffer, acetic acid-dissolved collagen I coatings substantially enhance MSC adhesion and spreading on both glass and polyacrylamide hydrogel substrates. When acetic acid was used for collagen coatings, even the low collagen concentration (1 *μ*g/ml) induced robust MSC spreading and nuclear YAP localization on both soft (3 kPa) and stiff (38 kPa) substrates. Depending on the solvent type, stiffness-dependent nuclear YAP translocation occurs at a different collagen concentration. Together, the results from this study validate the solvent type as an important parameter to consider when using collagen I as the biochemical ligand to support cell adhesion.

## ABBREVIATIONS

YAP: Yes-Associated Protein
ECM: Extracellular matrix
hMSCs: Human mesenchymal stem cells

## INTRODUCTION

Cells are surrounded by extracellular matrix (ECM) proteins composed of hundreds of proteins and glycoproteins^1^ that impart a myriad of physical and biological cues to the cells.^2^ Studies of cells *ex vivo* often require functionalization of surfaces with adhesive ECM proteins in order to permit cell attachment and growth.^3^ To enable mechanotransduction studies, polyacrylamide hydrogels with tunable stiffness have been widely used and require coating with ECM proteins to support cell adhesion.^4^

Type I collagen is an important structural component of ECM^1^ and is formed via self-assembly from tropocollagen units into small fibrils and then larger fibers.^5^ Given that type I collagen is the most abundant protein in the body^2^ and its relatively low cost compared to other ECM proteins, it is one of the most commonly used biochemical ligands for functionalizing hydrogel substrates to support cell adhesion.^4,6–11^ Previous mechanotransduction studies using collagen I coating have been plagued by varying the efficiency and heterogeneity, with coatings containing a mixture of long thin fibers^9^ or thick bundles of collagen aggregates.^10^ However, when interpreting the cell response, the distribution of collagen I coating was often not taken into consideration. Whether collagen I distribution on hydrogel substrates contributes to the observed changes in cell responses remains largely unknown.

As the structure and conformation of collagen I are known to be pH-dependent, we hypothesized that the pH of the solvent could alter the amount and distribution of collagen I coated. Collagen I is more soluble in acidic conditions, and increasing pH leads to self-assembly of collagen molecules into fiber structures.^12,13^ However, most conventional protocols for coating hydrogel substrates with collagen I use neutral solvents such as phosphate-buffered saline (PBS) (pH 7.4) or more basic solvents like HEPES (pH 8.5).^6^ This increased pH can lead to decreased solubility of collagen I in solution,^6^ which would subsequently contribute to the heterogeneity in collagen coating on the hydrogel substrates.

Cell adhesion is at the interface between the cell and the extracellular matrix and is the prerequisite for mechanotransduction and other cell fates. Varying ECM protein ligand density, for example, has been shown to modulate cell attachment, morphology,^7^ and stem cell mechanosensing via Yes-Associated Protein (YAP), a transcriptional regulator that translocates to the nucleus induced by stiff substrates^14^ or high ligand density.^15^ The structural conformation of collagen I has also been shown to influence mesenchymal stem cell (MSC) fate commitment between osteogenic and adipogenic lineages.^16^ We thus hypothesize that varying the pH and composition of solvents used for incorporating collagen I onto hydrogel substrates as biochemical cues will result in varying densities and distribution of collagen I, thereby altering cell attachment, spreading, and mechanotransduction. To test this hypothesis, we first analyzed the effects of varying solvent pH and type on protein coating efficiency and distribution on glass. Using human mesenchymal stem cells (hMSCs) as a model cell type, we then characterized MSC spreading on polyacrylamide hydrogels coated with collagen I using varying solvents including HEPES, PBS, and acetic acid. The effects of varying solvent types on stem cell spreading and YAP translocation were further evaluated by growing MSCs on collagen I-coated hydrogels with tunable stiffnesses (3 kPa or 38 kPa). Finally, the effects of varying solvent types on fibronectin coating and MSC response were also examined.

## RESULTS AND DISCUSSION

### Varying solvent pH and type alters collagen I coating distribution and efficiency on glass substrates

**FIG. 1.**
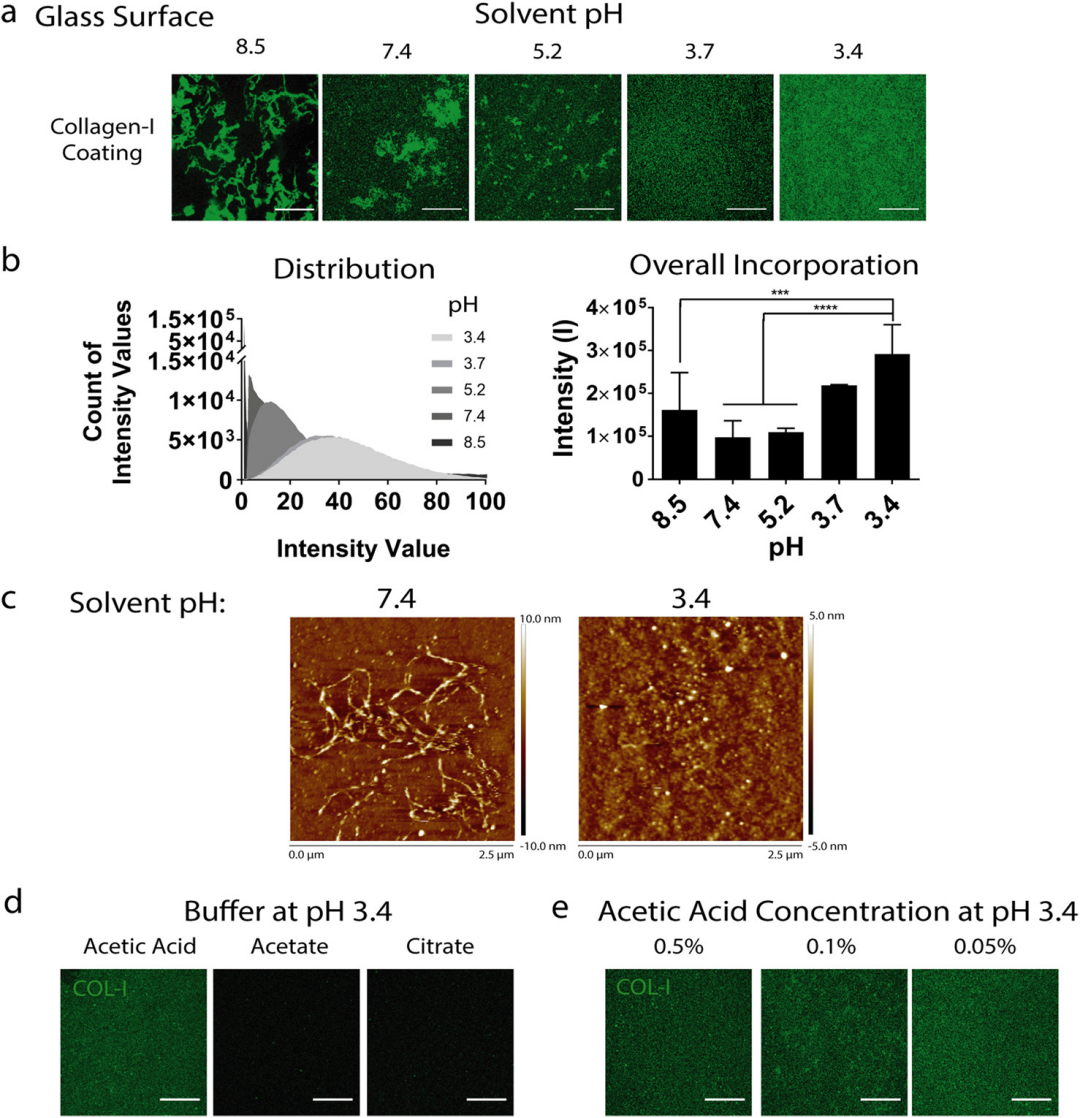
Varying the solvent pH and type alters collagen I coating distribution and efficiency on glass substrates. (a) Coatings on glass of 20 *μ*g/ml collagen I dissolved in acetic acid solvent adjusted to pH 8.5, 7.4, 5.2, 3.7, and 3.4, visualized through immunohistochemistry, (b) histograms (left) and plots of overall fluorescence intensity (right) for each condition, (c) atomic force microscopy of coatings with collagen I dissolved in solvent pH 7.4 and 3.4, (d) coatings of collagen I dissolved in acetic acid, acetate, or citrate, each buffered to pH 3.4, and (e) coatings of collagen I dissolved in different concentrations of acetic acid, each buffered to pH 3.4. Scale bars: 20 *μ*m, ****p < 0.0001.

While collagen I has been widely used to support cell adhesion for modulating various cell fates such as differentiation^3^ and mechanotransduction,^7–11^ the characterization of collagen coatings has been limited. For few studies that characterized collagen I coating on hydrogel surfaces, they generally showed heterogeneous distribution.^9,10^ To improve the homogeneity of collagen I coatings, we first tested if tuning the pH of the solvent used to dissolve collagen I could improve the solubility and homogeneity of collagen I. Although it is well known that low pH increases collagen I solubility,^12,13^ protocols for incorporating collagen I onto hydrogel substrates as biochemical cues often use solvents with neutral or basic pH.^6^ Therefore, we first tested how varying pH (3.4, 3.7, 5.2, 7.4, or 8.5) of the collagen I solution affects its solubility and coating on glass substrates, which should have high adsorption efficiency. At basic or neural pH (8.5 and 7.4), collagen I formed bundles and exhibited greater heterogeneity. Reducing pH increased the homogeneity, with acidic solvents leading to more homogeneous distributions of collagen I [Fig. 1(a)] and the highest amount of collagen I incorporated, as shown by the higher fluorescence intensity averages and histograms [Fig. 1(b)]. Increasing solvent acidity enhanced the overall intensity of collagen I staining, correlated with coating efficiency, and the normality of pixel distribution, correlated with coating distribution. To corroborate the results from fluorescence image analyses, we also used atomic force microscopy (AFM) to characterize the surface topographical cues of glass coated with collagen I dissolved in solvents with varying pH 7.4 or 3.4 [Fig. 1(c)]. Consistent with our fluorescence-image results, neutral pH (7.4) solvent resulted in more fibrous collagen formation, whereas acidic pH (3.4) showed more homogeneous punctate surface morphology. The peak height of the collagen fibers using pH 7.4 solvent is 10 nm, which is ∼2× of the peak height of collagen I punctae (5 nm) formed using solvent with pH 3.4, suggesting thicker collagen fiber bundle formation. Together, our results suggest that acidic pH (3.4) is preferable for enhancing collagen I homogeneity.

We then asked if varying the solvent composition without changing pH would impact the coating of collagen I. Holding pH constant at 3.4, we compared three solvents including acetic acid, acetate, and citrate [Fig. 1(d)]. Our results showed that collagen I incorporation efficiency depends not only on pH but also on the composition of the solvent. Among the three solvents tested, only acetic acid resulted in high levels of collagen I coating [Fig. 1(d)]. Using the optimal solvent pH (3.4) and solvent type (acetic acid), we further assessed how varying the concentration of acetic acid (0.05%–0.5%) impacts collagen I coating. Acetic acid supported a similar high level of collagen coating [Fig. 1(e)] even at the lowest tested concentration (0.05%). It is interesting to note that acetate buffer is composed of acetic acid plus sodium acetate yet results in much lower collagen I coating efficiency than acetic acid buffer. Our results show that collagen I dissolved in acetic acid led to higher collagen conjugation efficiency as compared to acetate and citrate. These results suggest that in addition to solvent pH, the choice of solvent composition matters too, possibly due to changes in the ionic strength and compositions.

### Varying the solvent type alters collagen I coating and cell attachment efficiency on both glass and polyacrylamide substrates

**FIG. 2.**
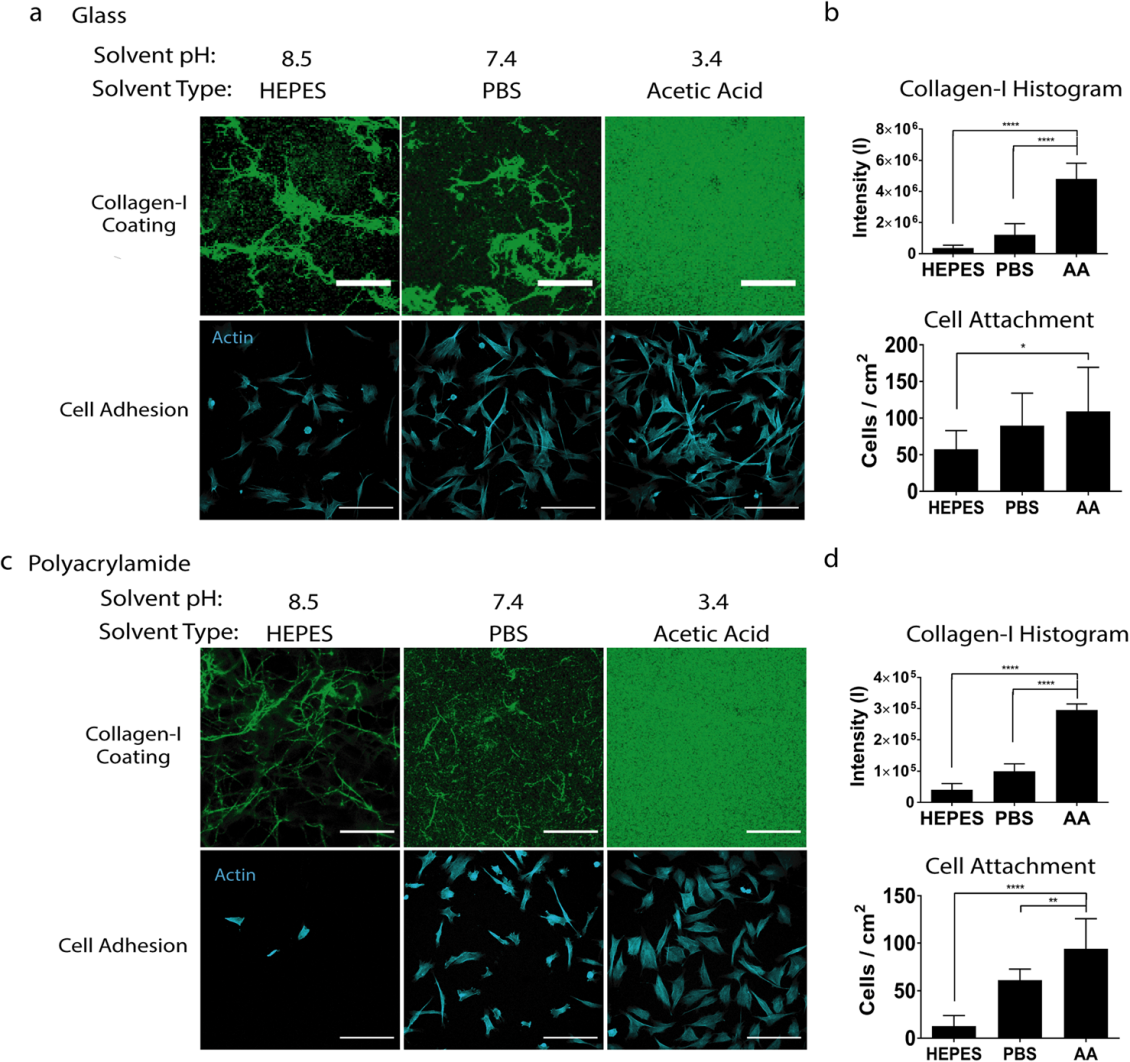
Varying the solvent type alters collagen I coating and human mesenchymal stem cell adhesion on both glass and polyacrylamide substrates. (a) Glass substrates and (c) soft polyacrylamide hydrogels (top) coated with 20 *μ*g/ml collagen I dissolved in HEPES (pH 8.5), PBS (pH 7.4), or acetic acid (pH 3.4) (Scale bars: 20 *μ*m) and (bottom) human mesenchymal stem cells seeded on each condition (Scale bars: 200 *μ*m); (b) quantification on glass and (d) polyacrylamide hydrogels of (top) collagen I distribution and (bottom) cell attachment. ****p < 0.0001.

Three of the most commonly used solvents for conjugating collagen I onto hydrogel substrates as a biochemical coating include HEPES (pH 8.5), PBS (pH 7.4), and acetic acid (pH 3.4). We compared these three solvents side-by-side to assess their effect on collagen coatings. Collagen I dissolved in HEPES buffer, as specified by common protocols in the field of mechanotransduction,^6^ led to a heterogeneous coating on glass with thick collagen bundle formation [Fig. 2(a)]. PBS resulted in similar heterogeneous collagen fiber formation. Only the acetic acid group exhibited an intense and homogeneous collagen I coating. Quantitative analyses of the fluorescence images showed much higher collagen I intensity in the acetic acid group compared to HEPES and PBS groups, indicating greater incorporation efficiency [Fig. 2(b)]. To assess the effect on cell attachment efficiency, MSCs were plated on these substrates. Consistent with the trend observed with collagen I coating efficiency, we found that acetic acid led to the best cell attachment efficiency, although the differences between the groups were less than the differences in collagen I coating [Fig. 2(b)]. These results indicate that the solvent type significantly impacts collagen I coating efficiency on glass substrates and cell attachment efficiency. The question we asked in this paper is the effect of initial collagen I coating on cell responses. We recognize that cells can deposit their own ECM over time, in addition to the initial collagen coating we presented. To minimize the confounding effects from ECM proteins secreted by the cells, we intentionally chose to perform our experiments and analyses at very early time points (6 h or less).

Given that most mechanotransduction studies use polyacrylamide hydrogel substrates, we further compared the effects of varying the solvent type on collagen I coatings on soft polyacrylamide hydrogel substrates (3 kPa). Acetic acid resulted in the highest collagen I coating homogeneity and cell attachment efficiency [Figs. 2(c) and 2(d)] on soft polyacrylamide hydrogels. Correspondingly, acetic acid conditions had the highest MSC attachment efficiency on polyacrylamide hydrogel substrates as well [Figs. 2(c) and 2(d)]. We further validated this attachment at an earlier time point (one hour) and observed a similar trend in cell attachment on soft polyacrylamide hydrogels (Fig. S1). To validate if the effects of the solvent type also depend on substrate compositions, we repeated the experiments of varying solvent types using two other commonly used substrates including polystyrene and polydimethylsiloxane (PDMS) (Fig. S2). We found the same trend: with acetic acid being most effective in achieving homogeneous collagen I coating with high efficiency. These results confirm acetic acid as the most effective solvent to achieve homogeneous and efficient collagen I coatings regardless of the substrate types. In the present study, collagen I was conjugated to the polyacrylamide surface via the heterofunctional bilinker sulfo-SANPAH, and this reaction is known to be pH sensitive, with higher efficiency at higher pH. To assess the effect of varying pH on protein conjugation efficiency, we have compared collagen I incorporation with polyacrylamide hydrogels with and without sulfo-SANPAH, using collagen I dissolved in acetic acid or PBS (neutral pH). When sulfo-SANPAH was used, we observed much higher and homogeneous collagen I incorporation using acetic acid as the solvent vs PBS (Fig. S3). In the absence of sulfo-SANPAH, an opposite trend was observed, with minimal collagen incorporation observed in collagen I dissolved in acetic acid.

### Varying the solvent type modulates collagen I coating and cell spreading on polyacrylamide hydrogels with tunable stiffnesses

**FIG. 3.**
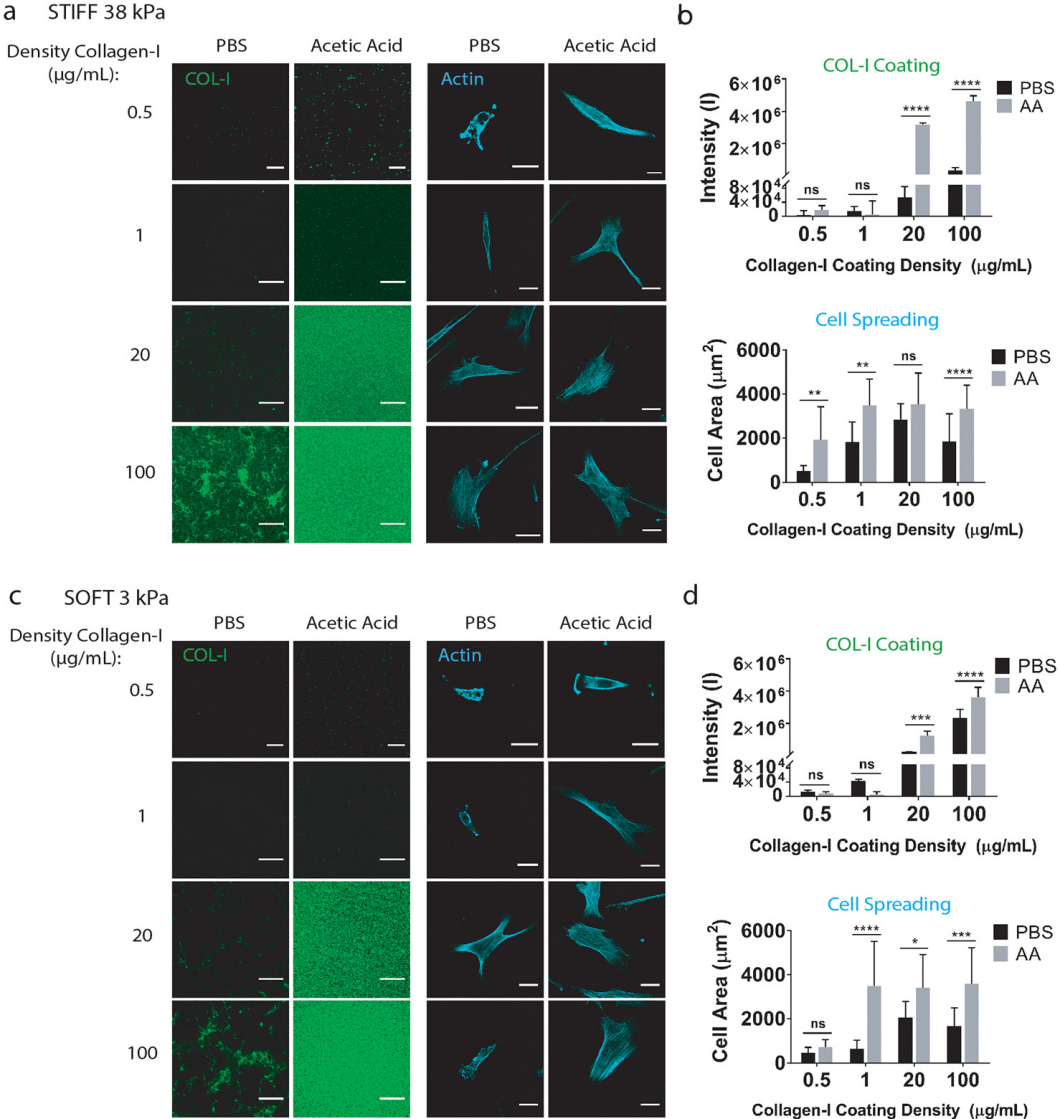
Varying the solvent type alters collagen I coating and cell spreading on polyacrylamide hydrogels with tunable stiffnesses and coating densities. Immunostaining of collagen I coating and F-actin (for visualizing cell morphology) expression by human MSCs on (a) stiff or (c) soft polyacrylamide hydrogels using PBS or acetic acid. Quantification of collagen I coating intensity and cell areas was characterized for both (b) stiff and (d) soft hydrogels. Green: collagen I; Scale bars: 20 *μ*m; Cyan: F-actin; Scale bars: 30 *μ*m. ****p < 0.0001.

Given mechanotransduction studies use hydrogels with tunable stiffnesses, we next characterized the effect of varying the solvent type (PBS vs acetic acid) on collagen I coatings and cell spreading on stiff (38 kPa) or soft (3 kPa) polyacrylamide hydrogels. For each solvent type, we also tested a broad range of collagen I concentrations (0.5, 1, 20, and 100 *μ*g/ml) used for coating to determine the minimal concentration needed for efficient collagen I coating. When PBS was used, only the highest concentration of collagen I solution (100 *μ*g/ml) led to noticeable collagen I staining, with heterogeneous collagen I fiber formation [Figs. 3(a) and 3(b)]. In contrast, acetic acid allowed homogeneous and high collagen I coating using a much lower concentration (20 *μ*g/ml), with homogeneous collagen I distribution. Similar trends were observed on both stiff (38 kPa) and soft (3 kPa) hydrogel substrates [Figs. 3(a) and 3(c)].

Cell morphology is an important indicator of how cells respond to substrate stiffness. Immunostaining of F-actin shows acetic acid supported more extensive cell spreading than PBS on both soft and stiff hydrogels, with a significantly higher cell area as shown by the quantification [Figs. 3(b) and 3(d)]. This is consistent with previous reports that cell spreading depends on ligand density.^7,22^ However, the difference in the cell area between two solvent types is much less than the differences in the collagen I intensity, especially when using a higher concentration of collagen (20 or 100 *μ*g/ml). These results suggest that once the threshold of collagen I coating density needed for supporting cell spreading is reached, a further increase in the collagen concentration would not further increase cell spreading. Based on our results, using acetic acid requires a much lower collagen concentration to reach such a threshold than using PBS as the solvent. For soft hydrogels, 1 *μ*g/ml collagen I in acetic acid was sufficient to reach the maximum cell spreading, whereas 20 *μ*g/ml collagen I in PBS was needed to reach the maximum cell spreading. The observed differences in cell spreading may also be in part due to the differences in collagen distribution between PBS and acetic acid. Such differences in distribution of collagen I can induce changes in accessibility of binding domains to which the cells adhere through integrin receptors on their surface.

We compared cell responses on stiff hydrogels (38 kPa) [Figs. 3(a) and 3(b)] with cell responses on soft hydrogels (3 kPa) [Figs. 3(c) and 3(d)]. Similar effects of varying solvent types on collagen I incorporation were observed regardless of hydrogel stiffnesses. Interestingly, collagen I dissolved in acetic acid produced coatings that enabled robust cell spreading even with doses as low as 1 *μ*g/ml. We had to decrease the collagen I concentration to 0.5 *μ*g/ml in order to see a decrease in cell spreading. This could offer a more resource- and cost-effective coating method for applications that requires efficient cell attachment and spreading.

### Varying solvent for collagen I coating impacts YAP translocation in hMSCs

**FIG. 4.**
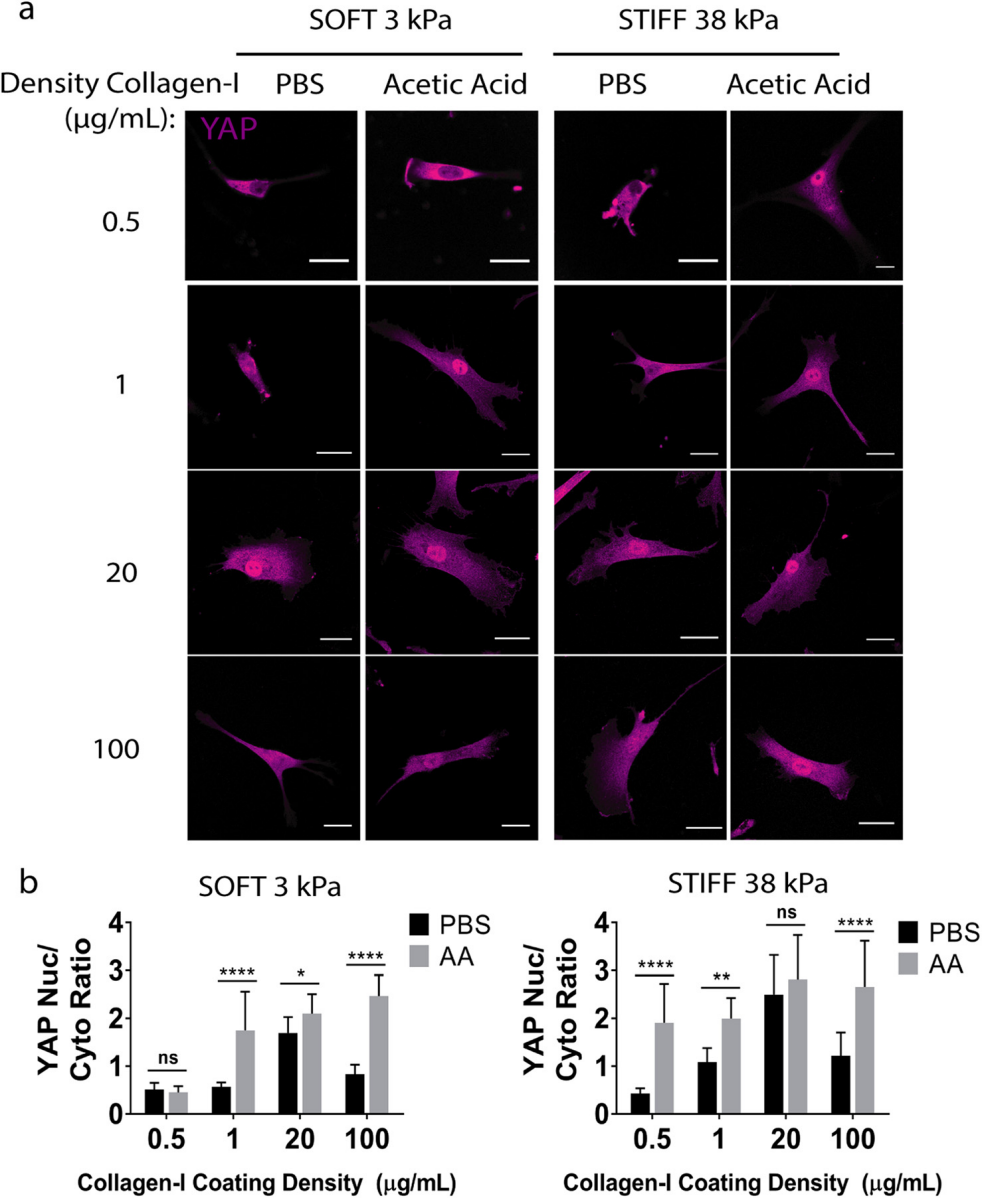
The solvent type alters YAP translocation of human mesenchymal stem cells at various collagen densities and stiffnesses. (a) YAP localization on (left) soft and (right) stiff hydrogels coated with collagen-I dissolved in PBS or acetic acid (magenta: YAP; Scale bars: 30 *μ*m) and (b) quantification of YAP localization for (left) soft and (right) stiff hydrogels. ****p < 0.0001.

Cell spreading is linked to mechanotransduction and has been shown to correlate with the translocation of transcriptional regulator YAP.^14^ We next assessed the effect of varying solvent on how stem cells sense the substrate stiffness by visualizing YAP localization through immunostaining. YAP is known to be sequestered to the cytoplasm when cells sense the substrate as soft and translocates to the nucleus if the cells sense the substrate as stiff. We found that the effects of varying the solvent type on YAP localization mirrored the trend observed for cell spreading [Figs. 4(a) and 4(b)]. When acetic acid was used as the solvent, even collagen I concentrations as low as 1 *μ*g/ml resulted in nuclear YAP localization on both soft and stiff substrates. Only when the collagen I concentration was further reduced to 0.5 *μ*g/ml, we observed cytoplasmic YAP localization in hMSCs on soft substrates, but not on stiff substrates. In contrast, PBS requires higher concentrations of collagen I (20 *μ*g/ml) to induce YAP nuclear localization, but a further increase in the collagen I concentration to 100 *μ*g/ml led to decreased YAP localization. The same trend was observed regardless of substrate stiffness, suggesting that biochemical cues override substrate stiffness and dominate the mechanotransduction.

To assess whether the solvent type-dependent YAP localization is associated with changes in the intensity and distribution of collagen coating, immunostaining of collagen I was performed after hMSCs were seeded and adhered to the surface. PBS coating resulted in heterogeneous collagen coating and less cell spreading, accompanied by cytoplasmic YAP (Fig. S4). Acetic acid, on the other hand, resulted in homogeneous collagen I coating and more cell spreading, accompanied by nuclear YAP (Fig. S4). Our results showed that low pH solvent (i.e., pH 3.4) led to more homogeneous distribution of collagen (Fig. 1). Conversely, increasing pH increases the heterogeneity and collagen bundle formation. The structural features of collagen affect cell spreading and YAP translocation, with more uniform coating leading to enhanced cell spreading and more nuclear YAP translocation. Given that YAP is a mechanical rheostat of the cell, our results demonstrate that varying the solvent type used for collagen I coating can directly alter how cells interpret the substrate stiffness. These results suggest that the solvent type can be harnessed as another tool to directly impact cell mechanotransduction, which in turn alters cell responses such as differentiation, proliferation, and migration.

### Varying the solvent type for fibronectin coating does not impact cell spreading and YAP localization

**FIG. 5.**
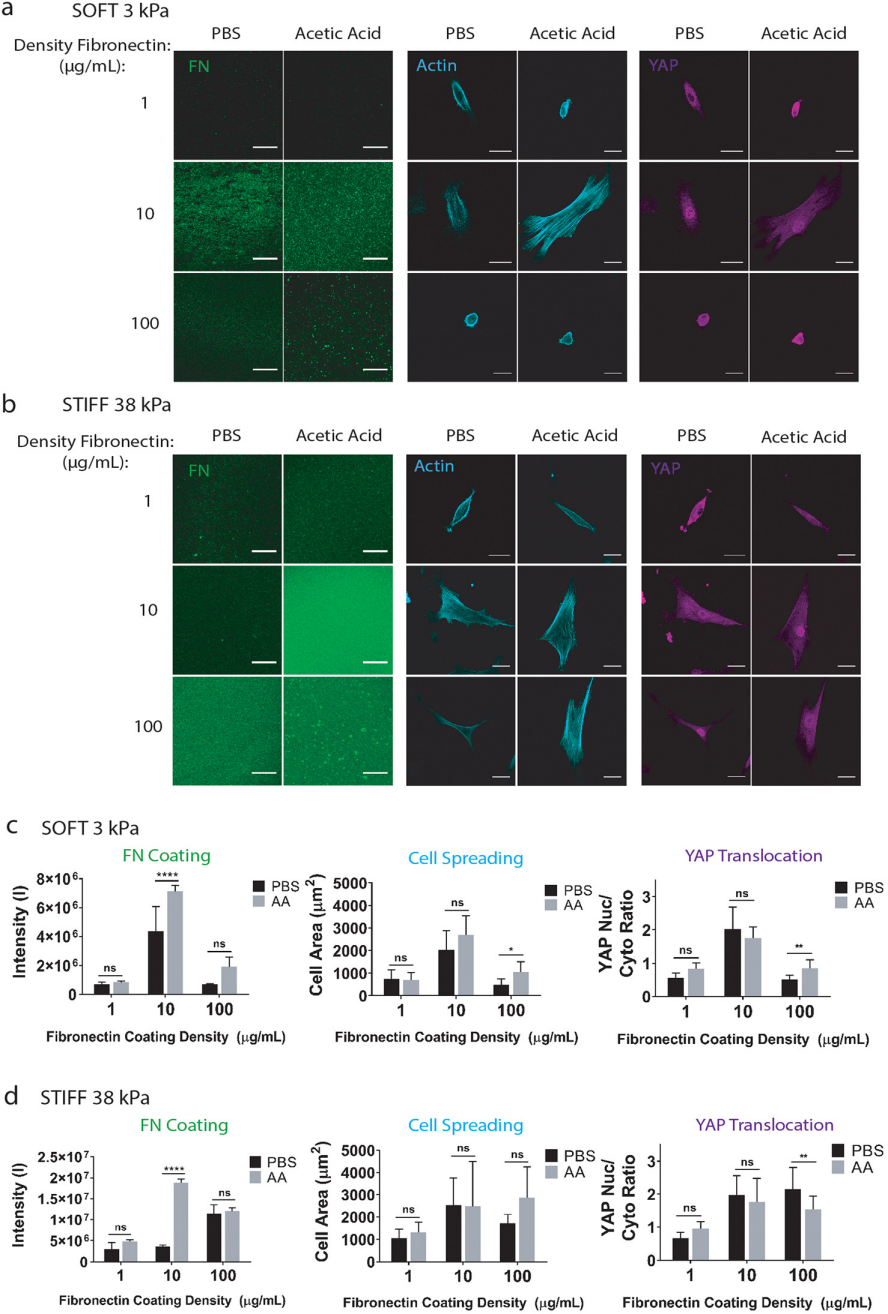
Fibronectin coatings in acetic acid vs PBS lead to similar cell spreading and YAP translocation. (a) Soft and (b) stiff polyacrylamide hydrogels coated with 1, 10, or 100 *μ*g/ml fibronectin dissolved in PBS or acetic acid and stained for (left) fibronectin to visualize the coating (green: fibronectin; Scale bars: 20 *μ*m) or seeded with the human mesenchymal stem cells and stained for (middle) F-actin (cyan: F-actin; Scale bars: 30 *μ*m) or (right) YAP (magenta: YAP; Scale bars: 30 *μ*m), and quantification of (c) soft and (d) stiff conditions. ****p < 0.0001.

In addition to collagen I, fibronectin is another widely used biochemical coating for mechanotransduction studies.^6,14,23–26^ Therefore, we also tested the effect of varying the solvent type (PBS vs acetic acid) on fibronectin coating and YAP localization using hMSCs. Unlike the case for collagen I, both solvent types resulted in homogeneous coating for fibronectin, most likely because the solubility of fibronectin is not pH sensitive. While acetic acid enhanced fibronectin coating on the stiff substrate at higher densities (10 *μ*g/ml or 100 *μ*g/ml), varying the solvent type does not impact hMSC cell spreading or YAP localization in response to changes in substrate stiffness and biochemical ligand density (Fig. 5). These results suggest that the effect of the solvent type on mechanosensing is highly dependent on the type of ECM and should be evaluated individually depending on the ECM of interest of different studies.

## CONCLUSION

In summary, we demonstrated that collagen I coating efficiency and distribution on hydrogel substrates depend on the choice of solvent pH and composition. Among the various commonly used solvents, acetic acid results in the most efficient collagen I coating with homogeneous distribution. Importantly, varying the solvent type can directly impact cell spreading and mechanotransduction of hMSCs, as shown by the changes in YAP translocation. Given the important role of mechanotransduction in various cell fates, our results suggest that the solvent type can be harnessed as a new tool to directly impact cell mechanotransduction and fates such as differentiation, proliferation and migration. Unlike collagen I, we further showed that fibronectin coating is less sensitive to solvent pH and type. Together, these results suggest that the effect of the solvent type on mechanotransduction is highly dependent on the type of ECM and should be evaluated individually depending on the ECM of interest for each study.

## METHODS

### Fabrication of polyacrylamide hydrogel substrate

Polyacrylamide hydrogels were fabricated as we have reported^15,17^ by adapting a previous protocol^6,18^ to incorporate primary amine end groups, for the purpose of enhancing protein conjugation efficiency. In brief, 2-aminoethyl methacrylate (Aldrich 516155, 15 mM in de-ionized water) was added to hydrogel precursor solution containing acrylamide (Sigma A4058,40% (v/v)) and N,N′-methylenebisacrylamide [Sigma M1533, 2% (v/v)]. Soft or stiff hydrogels were fabricated by maintaining the acrylamide concentration constant [8% (v/v)] while varying the concentration of bis-acrylamide [0.08% or 0.48% (v/v)], resulting in hydrogels with stiffnesses of 3 and 38 kPa, respectively, as we previously reported.^15^ To initiate photocrosslinking, photoinitiator 2-Hydroxy-1-[4–(2-hydroxyethoxy) phenyl]-2-methyl-1-propanone [Irgacure 2959, Ciba, 0.05% (w/v)] was used. Hydrogel precursor solution (65 *μ*l) was loaded between two round glass coverslips (15 mm in diameter) and exposed to ultraviolet light (365 nm, 4 mW/cm^2^, 5 min) to form a hydrogel substrate with a thickness of ∼370 *μ*m. The hydrogel surface was then modified with sulfo-SANPAH (Life Technologies 22589, 0.83 mg/ml in PBS) and exposed to light (365 nm, 4 mW/cm^2^, 5 min).

### Fabrication of PDMS substrates

PDMS substrates were prepared using a 1:10 mixture of curing agent to PDMS base (Dow Stylgard 184 Silicone Elastomer). After whisking vigorously for 10 min, the solution was dessicated for 60 min and then cured for 2 h at 60 °C.

### Solvent preparation

0.1% acetic acid solutions were prepared at pH 3.4, 3.7, 5.2, 7.4, or 8.5; 0.5% or 0.05% Acetic acid at pH 3.4; Acetate or Citrate solvents at pH 3.4; PBS at pH 7.4; or HEPES at pH 8.5. pH was tuned using concentrated NaOH.

### Collagen I incorporation

Glass coverslips, polystyrene, PDMS, and polyacrylamide hydrogels, all 15 mm circles, were coated with collagen I under sterile conditions. Acetic acid solvents were made at 0.05%, 0.1%, and 0.5%. Acetate was made by combining 0.1 M Acetic acid and 0.1 M Sodium acetate at 46.3% and 3.7%, respectively, and buffered to pH 3.4. Citrate was made by combining 0.1 M Citric acid and 0.2 M Dibasic sodium phosphate at 35.9% and 14.1%, respectively. Collagen I (rat tail, Corning CB40236) was kept on ice and diluted immediately following substrate synthesis in the appropriate solvent (0.1% Acetic acid at pH 3.4, 3.7, 5.2, 7.4, or 8.5; 0.5% or 0.05% Acetic acid at pH 3.4; Acetate or Citrate solvents at pH 3.4; PBS at pH 7.4; or HEPES at pH 8.5) for a final concentration of 20 *μ*g/ml for most studies and of 0.5, 1, 20, or 100 *μ*g/ml in polyacrylamide studies to test the effect of collagen coating on cell response. Substrates were washed with PBS, covered with 150 *μ*l of collagen I solution, and incubated overnight at 37 °C.

### Cell culture

Bone marrow-derived human mesenchymal stem cells (Lonza) were cultured in growth medium composed of Dulbecco's Modified Eagle Medium (Gibco), fetal bovine serum (10% v/v, Gibco), penicillin-streptomycin (1% v/v, ThermoFisher Scientific), and recombinant human fibroblast growth factor-basic (10 ng/ml, Peprotech). For all cell studies, passage 6 hMSCs were plated at 5000 cells/cm^2^ onto the hydrogels and cultured 6 h before being analyzed by immunofluorescence staining. Ethics approval is not required for this study.

### Immunofluorescence staining

Cells were fixed using 4% paraformaldehyde/PBS for 15 min at room temperature, washed three times with washing solvent (0.1% Tween-20/PBS, 5 min), and permeabilized with 1% Triton X-100/PBS for 30 min. Samples were incubated in blocking solvent (3% bovine serum albumin, 2% goat serum in PBS) for 30 min. For YAP staining, samples were incubated with 1:300 mouse anti-YAP (Santa Cruz Biotechnology, sc-101199) overnight at 4 °C on a shaker. After washing, samples were incubated with 1:300 Alexa 488 Goat-antimouse (Invitrogen A11001) for 1 h at room temperature on a shaker. Cell nucleus counter stain was performed using Hoechst nuclear stain (Cell Signaling Technology 4082S, 2 ug/ml). Actin staining was performed using the stain rhodamine-phalloidin (Sigma P1951). Samples were washed with washing solvent (three times, 5 min per wash) before being imaged using a confocal microscope (10× air, 40× oil, or 100× oil immersion, Leica SP8 confocal system). To visualize collagen I coatings, substrates were washed with PBS and then immediately stained following the above protocol with blocking solvent, 1:100 primary rabbit anticollagen I antibody (Abcam ab34710), and Alexa 488 Goat-antirabbit (Invitrogen A11034). All images were processed using open-source Fiji software.^19,20^

### Image analyses

To quantify protein incorporation to the surface of the hydrogels, two methods were employed. First, we measured the pixel intensities of representative images and plotted the results in histograms to assess the distribution of the protein coating. Second, we compared the intensities of the different conditions, using the area under the curve of these histograms, to characterize the total incorporation. For quantification of collagen I fluorescence intensity, an average of 9 fields of view on 3 independent substrates were assessed for pH dose experiments [Fig. 1(b)], an average of 13 fields of view on 3 independent substrates for buffer experiments (Fig. 2), and an average of 5 fields of view on 3 independent substrates for collagen dose experiments (Fig. 3). For assessing cell attachment, an average of 50 fields of view from 3 independent substrates were taken, cells were counted, and counts were averaged for 1 h time points (Fig. S1) and 10 fields of view from 3 independent substrates for 6 h time points (Fig. 2). To characterize YAP localization in a quantitative manner, we employed a method^21^ which reports the ratio of nuclear YAP intensity vs cytoplasm YAP intensity. In brief, a region of interest (ROI) in the nucleus and a region of interest of equal area in the cytoplasm immediately adjacent to the nucleus were selected. The nuclear region was defined using Hoechst staining. The fluorescence intensity of YAP staining within the nucleus ROI and the cytoplasm ROI was then quantified. The results are reported as the ratio of fluorescence intensity within nucleus vs fluorescence intensity in cytoplasm for an average of 22 cells imaged at high magnification from 3 independent substrates [Fig. 4(b)]. The cell area was measured by thresholding the background and selecting the cell perimeter, for an average of 20 cells from 3 independent substrates (Fig. 3).

### Atomic force microscopy

Topography of collagen fibrils was assessed using an atomic force microscope (AFM), conducted on a Bruker BioScope Resolve (Bio-AFM, Bruker Nano) and ScanAsyst-Fluid probe. The probe has a silicon nitride triangular tip with radium of 20 nm and a 70 *μ*m cantilever with a spring constant of 0.7 N/m. Before imaging, cover slips coated with collagen solutions in acetic acid or PBS, with varying concentrations, were glued to Petri dishes that can be mounted on Bio-AFM. The collagen fibrils were imaged using a Peak Force Quantitative Nanoscale Mechanical (Peak Force QNM) module and in tapping mode to obtain maps with dimensions of 2.5 *μ*m × 2.5 *μ*m.

### Statistical analyses

Data are presented as mean ± standard deviations. For comparisons, data were analyzed with GraphPad Prism using one-way Analysis of Variance (ANOVA) by Tukey's multiple comparison test or two-way ANOVA by Bonferroni's multiple comparison test. Confidence intervals were kept at 95%, and P-values less than 0.05 were considered statistically significant.

## SUPPLEMENTARY MATERIAL

See the supplementary material for additional data of collagen I incorporation on polystyrene and PDMS substrates, the role of sulfo-SANPAH in the conjugation of collagen I in neutral and acidic solvents, assessment of cell attachment after one hour, and costaining of collagen I and YAP.

## References

[1] R. Hynes and A. Naba , “ Overview of the matrisome–an inventory of extracellular matrix constituents and functions,” Cold Spring Harbor Perspect. Biol. 4(1), a004903 (2012). 10.1101/cshperspect.a004903 PMC3249625 21937732

[2] J. D. Humphrey , E. R. Dufresne , and M. A. Schwartz , “ Mechanotransduction and extracellular matrix homeostasis,” Nat. Rev. Mol. Cell Biol. 15, 802–812 (2014). 10.1038/nrm3896 25355505 PMC4513363

[3] M. Kosovsky , “ Culture conditions and ECM surfaces utilized for the investigation of stem cell differentiation,” Corning Review CLS-DL-CC-041-A4, 2013.

[4] R. K. Das , “ Harnessing cell-material interaction to control cell fate: Design principle of advanced functional hydrogel materials,” J. Chem. Sci. 129, 1807–1816 (2017). 10.1007/s12039-017-1387-y

[5] C. Domene , C. Jorgensen , and S. W. Abbasi , “ A perspective on structural and computational work on collagen,” Phys. Chem. Chem. Phys. 18, 24802–24811 (2016). 10.1039/C6CP03403A 27711449

[6] J. R. Tse and A. J. Engler , “ Preparation of hydrogel substrates with tunable mechanical properties,” Curr. Protoc. Cell Biol. 47, 10.16.1–10.16.16 (2010). 10.1002/0471143030.cb1016s47 20521229

[7] A. Engler , L. Bacakova , C. Newman , A. Hategan , M. Griffin , and D. Discher , “ Substrate compliance versus ligand density in cell on gel responses,” Biophys. J. 86, 617–628 (2004). 10.1016/S0006-3495(04)74140-5 14695306 PMC1303831

[8] A. J. Engler , S. Sen , H. L. Sweeney , and D. E. Discher , “ Matrix elasticity directs stem cell lineage specification,” Cell 126, 677–689 (2006). 10.1016/j.cell.2006.06.044 16923388

[9] J. H. Wen , L. G. Vincent , A. Fuhrmann , Y. S. Choi , K. C. Hribar , H. Taylor-Weiner , S. Chen , and A. J. Engler , “ Interplay of matrix stiffness and protein tethering in stem cell differentiation,” Nat. Mater. 13, 979–987 (2014). 10.1038/nmat4051 25108614 PMC4172528

[10] B. Trappmann , J. E. Gautrot , J. T. Connelly , D. G. T. Strange , Y. Li , M. L. Oyen , M. A. Cohen Stuart , H. Boehm , B. Li , V. Vogel , J. P. Spatz , F. M. Watt , and W. T. S. Huck , “ Extracellular-matrix tethering regulates stem-cell fate,” Nat. Mater. 11, 642–649 (2012). 10.1038/nmat3339 22635042

[11] L. Valon , A. Marin-Llaurado , T. Wyatt , G. Charras , and X. Trepat , “ Optogenetic control of cellular forces and mechanotransduction,” Nat. Commun. 8, 14396 (2017). 10.1038/ncomms14396 28186127 PMC5309899

[12] T. Hayashi and Y. Nagai , “ Effect of pH on the stability of collagen molecule in solution,” J. Biochem. 73, 999–1006 (1973). 10.1093/oxfordjournals.jbchem.a130184 4578804

[13] B. R. Williams , R. A. Gelman , D. C. Poppke , and K. A. Piez , “ Collagen fibril formation,” J. Biol. Chem. 253, 6578–6585 (1978). 28330

[14] S. Dupont , L. Morsut , M. Aragona , E. Enzo , S. Giulitti , M. Cordenonsi , F. Zanconato , J. Le Digabel , M. Forcato , S. Bicciato , N. Elvassore , and S. Piccolo , “ Role of YAP/TAZ in mechanotransduction,” Nature 474, 179–183 (2011). 10.1038/nature10137 21654799

[15] A. E. Stanton , X. Tong , S. Lee , and F. Yang , “ Biochemical ligand density regulates yes-associated protein translocation in stem cells through cytoskeletal tension and integrins,” ACS Appl. Mater. Interfaces 11, 8849–8857 (2019). 10.1021/acsami.8b21270 30789697 PMC6881158

[16] J. Mauney and V. Volloch , “ Progression of human bone marrow stromal cells into both osteogenic and adipogenic lineages is differentially regulated by structural conformation of collagen I matrix via distinct signaling pathways,” Matrix Biol. 28, 239–250 (2009). 10.1016/j.matbio.2009.04.003 19375503 PMC6817339

[17] S. Lee , A. E. Stanton , X. Tong , and F. Yang , “ Hydrogels with enhanced protein conjugation efficiency reveal stiffness-induced YAP localization in stem cells depends on biochemical cues,” Biomaterials 202, 26–34 (2019). 10.1016/j.biomaterials.2019.02.021 30826537 PMC6447317

[18] R. J. Pelham and Y. Wang , “ Cell locomotion and focal adhesions are regulated by substrate flexibility,” Proc. Natl. Acad. Sci. 94, 13661–13665 (1997). 10.1073/pnas.94.25.13661 9391082 PMC28362

[19] J. Schindelin , I. Arganda-Carreras , E. Frise , V. Kaynig , M. Longair , T. Pietzsch , S. Preibisch , C. Rueden , S. Saalfeld , B. Schmid , J. Y. Tinevez , D. J. White , V. Hartenstein , K. Eliceiri , P. Tomancak , and A. Cardona , “ Fiji: An open-source platform for biological-image analysis,” Nat. Methods 9, 676–682 (2012). 10.1038/nmeth.2019 22743772 PMC3855844

[20] J. Schindelin , C. T. Rueden , M. C. Hiner , and K. W. Eliceiri , “ The ImageJ ecosystem: An open platform for biomedical image analysis,” Mol. Reprod. Dev. 82, 518–529 (2015). 10.1002/mrd.22489 26153368 PMC5428984

[21] A. Elosegui-Artola , R. Oria , Y. Chen , A. Kosmalska , C. Perez-Gonzalez , N. Castro , C. Zhu , X. Trepat , and P. Roca-Cusachs , “ Mechanical regulation of a molecular clutch defines force transmission and transduction in response to matrix rigidity,” Nat. Cell Biol. 18, 540–548 (2016). 10.1038/ncb3336 27065098

[22] C. Gaudet , W. A. Marganski , S. Kim , C. T. Brown , V. Gunderia , M. Dembo , and J. Y. Wong , “ Influence of type I collagen surface density on fibroblast spreading, motility, and contractility,” Biophys. J. 85, 3329–3335 (2003). 10.1016/S0006-3495(03)74752-3 14581234 PMC1303610

[23] S. R. Peyton and A. J. Putnam , “ Extracellular matrix rigidity governs smooth muscle cell motility in a biphasic fashion,” J. Cell Physiol. 204, 198–209 (2005). 10.1002/jcp.20274 15669099

[24] A. Elosegui-Artola , E. Bazellieres , M. D. Allen , I. Andreu , R. Oria , R. Sunyer , J. J. Gomm , J. F. Marshall , J. L. Jones , X. Trepat , and P. Roca-Cusachs , “ Rigidity sensing and adaptation through regulation of integrin types,” Nat. Mater. 13, 631–637 (2014). 10.1038/nmat3960 24793358 PMC4031069

[25] W. J. Hadden , J. L. Young , A. Holle , M. L. McFetridge , D. Y. Kim , P. Wijesinghe , H. Taylor-Weiner , J. H. Wen , A. R. Lee , K. Bieback , B. Vo , D. D. Sampson , B. F. Kennedy , J. P. Spatz , A. J. Engler , and Y. S. Choi , “ Stem cell migration and mechanotransduction on linear stiffness gradient hydrogels,” Proc. Natl. Acad. Sci. 114, 5647–5652 (2017). 10.1073/pnas.1618239114 28507138 PMC5465928

[26] G. Nardone , J. Oliver-De La Cruz , J. Vrbsky , C. Martini , J. Pribyl , P. Skladal , M. Pesl , G. Caluori , S. Pagliari , F. Martino , Z. Maceckova , M. Hajduch , A. Sanz-Garcia , N. M. Pugno , G. B. Stokin , and G. Forte , “ YAP regulates cell mechanics by controlling focal adhesion assembly,” Nat. Commun. 8, 15321 (2017). 10.1038/ncomms15321 28504269 PMC5440673

